# A phase II trial to assess the activity of gemcitabine and docetaxel as first line chemotherapy treatment in patients with unresectable leiomyosarcoma

**DOI:** 10.1186/s13569-015-0029-8

**Published:** 2015-05-16

**Authors:** Beatrice Seddon, Michelle Scurr, Robin L Jones, Zoe Wood, Cerys Propert-Lewis, Cyril Fisher, Adrienne Flanagan, Jonanthan Sunkersing, Roger A’Hern, Jeremy Whelan, Ian Judson

**Affiliations:** Sarcoma Unit, University College Hospital, 1st Floor Central, 250 Euston Road, London, NW1 2PG UK; Sarcoma Unit, Royal Marsden Hospital, Fulham Road, London, SW3 6JJ UK; Department of Histopathology, Royal National Orthopaedic Hospital, Brockley Hill, Stanmore, Middlesex HA7 4LP UK; ICR Clinical Trials and Statistics Unit, Institute of Cancer Research, 15 Cotswold Road, Sutton, SM2 5NG UK

**Keywords:** Gemcitabine, Docetaxel, Leiomyosarcoma

## Abstract

**Background:**

Gemcitabine and docetaxel have been shown to be active in pre-treated relapsed leiomyosarcoma. This study investigated the combination as first line treatment in patients with unresectable locally advanced/metastatic leiomyosarcoma.

**Methods:**

Patients received gemcitabine 900 mg/m^2^ days 1 and 8, and docetaxel 100 mg/m^2^ day 8, administered 3-weekly for up to 8 cycles, with GCSF support on days 9–15. Patients who had received previous radiotherapy were treated at 75% dose. Patients were evaluated for response by RECIST 1.0 after cycles 2, 4, 6 and 8, and 3-monthly after completing treatment.

**Results:**

Forty-four patients were evaluable for response. Eligible patients had histologically proven leiomyosarcoma of the uterus (54.5%) or other sites (45.5%). Thirty-nine patients (84.4%) had metastatic disease, and 5 (15.6%) had locally advanced disease. Six patients (13.6%) had grade 1 disease, and 23 (75%) had grade 2/3 disease. All patients had demonstrated disease progression prior to trial entry. Responses were as follows: partial response 11 (25.0%), stable disease (confirmed) 16 (36.6%), stable disease (unconfirmed) 7 (15.9%), progressive disease 10 (22.7%). Median progression-free survival and overall survival were 7.1 months (95% CI 5.7–8.3) and 17.9 months (95% CI 10.6–25.2), respectively. Progression free rates at 3 and 6 months were 70.5% (95% CI 56.7–84.2%) and 59.1% (95% CI 44.3–73.9%).

**Conclusions:**

This study demonstrates gemcitabine and docetaxel to be active in locally advanced/metastatic leiomyosarcoma in the first line setting. Further investigation comparing with current standard therapies for leiomyosarcoma is warranted.

## Background

Leiomyosarcoma represents one of the commonest subtypes of soft tissue sarcoma (STS). It can arise at any anatomical location, but the commonest sites are the female gynaecological organs (26%), limbs (16%), gastrointestinal tract (14%), trunk (13%), and retroperitoneum (6%) [1]. The age-standardised incidence of leiomyosarcoma in England for 2007–2009 was 9/million, with 470 cases in 2009 [1].

Outcomes for locally advanced unresectable or metastatic STS are poor, with a median OS of 12 months [2]. Chemotherapy is usually the initial treatment, and for many years the first line treatment has been single agent doxorubicin or combination doxorubicin and ifosfamide [3]. Most recently a prospective randomised phase III study has shown that while combination doxorubicin chemotherapy is associated with a longer PFS compared with doxorubicin alone, there was no difference in median OS between the two groups, at 14.3 and 12.8 months, respectively [4]. As yet, no other chemotherapy regimen has proved to be superior to doxorubicin-based regimens. However, for more than a decade there has been increasing use of the combination of fixed dose rate gemcitabine with docetaxel, which has shown encouraging activity in leiomyosarcoma, both uterine [5–7] and non-uterine [7, 8], and in STS more generally [8–10]. Fixed-dose rate gemcitabine refers to infusing the gemcitabine at a rate that maintains the plasma concentration of gemcitabine at levels that optimise its conversion into gemcitabine diphosphate (dFdCDP) and triphosphate (dFdCTP). The conversion process is saturable, hence the benefit of prolonged infusion. dFdCDP is an inhibitor of ribonucleotide reductase, and dFdCTP is incorporated into deoxyribonucleic acid (DNA) causing strand termination during DNA replication. Preclinical data indicate that maintaining the dFdCTP concentration at ≥20 μmol/l optimises tumour cell killing in vivo [11, 12]. Pharmacokinetic studies from a phase II study in leiomyosarcoma have shown that using fixed dose rate gemcitabine (10 mg/m^2^/min) increased the duration of time that dFdCTP remained above the threshold for incorporation into DNA, as compared with bolus gemcitabine [8].

The purpose of this study was to confirm the response rates observed in the previous studies of gemcitabine and docetaxel in STS, and to limit the cohort to leiomyosarcoma in the first line setting.

## Methods

### Patient eligibility

Patients with advanced unresectable leiomyosarcoma, of uterine origin or other sites of origin were eligible for this phase II study. All patients were recruited at either of two specialist sarcoma units. Other eligibility criteria were as follows: one or more sites of measurable disease according to RECIST 1.0 (which should not be within a previously irradiated field); no previous chemotherapy, but prior radiation allowed if completed >6 weeks prior to trial entry; adequate organ function (absolute neutrophil count ≥1 × 10^9^/l, platelet count ≥100 × 10^9^/l, total bilirubin ≤21 μmol/l, serum creatinine ≤130 μmol/l); performance status 0–2; no previous history of malignancy other than non-melanoma skin cancer or cervical carcinoma in situ; patients not pregnant or lactating; no active uncontrolled infection; no evidence of grade 3 or 4 peripheral neuropathy. All external histopathology was reviewed centrally by expert sarcoma pathologists at the two sarcoma centres.

All patients provided written informed consent form prior to study entry. Ethical permissions were given by both institutions’ Institutional Review Boards, and by the UK NHS Research Ethics Committee.

### Treatment

At study entry patients were evaluated with baseline history and clinical examination, blood tests and imaging [computerised tomography (CT)] scan of chest, abdomen and pelvis. During treatment, prior to each cycle patients were assessed for treatment-related toxicities, and underwent clinical examination, and blood tests (including weekly full blood counts, and urea, electrolytes and liver function) prior to each cycle. Disease response was evaluated by CT scans which were repeated prior to cycles 3, 5 and 7, and at the end of treatment, and then 3-monthly thereafter until disease progression.

Patients without a previous history of pelvic radiotherapy received fixed dose rate (10 mg/m^2^/min) gemcitabine 900 mg/m^2^ on days 1 and 8 given intravenously over 90 min, followed by docetaxel 100 mg/m^2^ on day 8 given intravenously over 60 min, for up to 8 cycles. Patients who had received previous pelvic radiotherapy were treated at 25% lower doses (gemcitabine 675 mg/m^2^ and docetaxel 75 mg/m^2^). Premedication used for docetaxel was dexamethasone 8 mg twice daily for 3 days starting the on day 7. Human recombinant granulocyte colony-simulating factor support was used with lenograstim (Granocyte™, Chugai Pharma UK Ltd) 263 μg daily subcutaneously on days 9–15.

Treatment toxicities were assessed according to the National Cancer Institute Common Toxicity Criteria version 2.0. If during a cycle platelets fell to <25 × 10^9^/l for >5 days and/or there was an episode of febrile neutropenia then doses of gemcitabine and docetaxel were reduced by 25% for all subsequent cycles. If on day 1 platelets were <100 × 10^9^/l or absolute neutrophil count (ANC) was <1.0 × 10^9^/l, then treatment was delayed for a week. If platelets or ANC had not recovered after a 2-week delay, then patients were removed from the study. If on day 8 ANC was 0.5–0.99 × 10^9^/l and/or platelets were 50–99 × 10^9^/l, then gemcitabine and docetaxel doses were reduced by 25%. If on day 8 ANC was <0.5 × 10^9^/l and/or platelets were <50 × 10^9^/l then gemcitabine and docetaxel were omitted for that cycle. If grade 3 or 4 neurotoxicity occurred, treatment was delayed by 1 week; if toxicity had resolved to ≤grade 2, then treatment was continued with a 25% dose reduction of docetaxel for all subsequent cycles. If bilirubin was >21 μmol/l, docetaxel was withheld for that cycle, and reinstated if bilirubin fell to ≤21 μmol/l. For any other grade 3 or 4 non-haematological toxicities, treatment should be delayed until resolution to ≤grade 2; if toxicity had not recovered after a 2-week delay, then patients were removed from the study.

All serious adverse events occurring during the treatment period and within 30 days after the end of the last protocol treatment were reported to the Research Ethics Committee.

### Disease response evaluation

All patients who received at least 2 cycles of study treatment were considered assessable for response. Response was assessed by tumour measurements made according to RECIST 1.0 [13]. The best overall response was the best response recorded from the start of treatment until disease progression/recurrence (taking as reference for progressive disease the smallest measurements recorded since the treatment started). To be assigned a status of partial response (PR) or complete response (CR), changes in tumour measurements were confirmed by repeat assessments performed no less than 4 weeks after the criteria for response were first met. In the case of stable disease (SD), follow-up measurements must have met the SD criteria at least once after study entry at a minimal interval defined in the protocol (in this case after 2 cycles of treatment).

### Statistical design

This was a phase II trial designed to have a 90% chance of concluding that the study treatment was effective if its true response rate was ≥35%, but with only a 5% chance of concluding it was effective if the response rate was ≤15%.

The trial had a Simon two-stage accrual design with an early stopping rule in the event that the study treatment demonstrated insufficient activity. Nineteen patients were to be recruited in the first stage, and 25 patients in the second stage, giving a possible total of 44 patients. If there were fewer than four responses in the first stage, the trial would be stopped early. If the study was completed, but there were fewer than 11 responses overall, then the study treatment would be considered inactive. If there were ≥4 responses in the first 19 patients and a total of ≥11 responses overall (≥25% response rate) then it would be concluded that the study treatment has sufficient activity to warrant further investigation in clinical trials.

The primary endpoint of the study was the objective response rate (ORR). Secondary end points were PFS, OS, and toxicity. The proportions of patients surviving and progression free during follow-up were calculated using the Kaplan–Meier method, and median follow-up was calculated using the reverse Kaplan–Meier method.

## Results

### Patient characteristics

Between August 2004 and December 2007, 45 patients were enrolled from two sarcoma units in London, UK. One patient was excluded from the analysis as she only received one cycle of chemotherapy and so was considered non-evaluable for response (this patient deteriorated rapidly after cycle 1 and died). No patients had received prior adjuvant chemotherapy. Patient and disease characteristics are summarised in Table 1.

**Table 1:** Patient characteristics (n = 44)

	N	%
Age (years)		
Median	53	
Range	31–73	
Sex		
Male	5	11.4
Female	39	88.6
Primary site of disease		
Uterus	24	54.5
Retroperitoneum	7	15.9
Other	7	15.9
Extremity	2	4.5
Vascular	2	4.5
Thoracic	1	2.3
Not known	1	2.3
Disease type		
Locally advanced	5	11.4
Metastatic	39	88.6
Tumour grade		
Grade 1	6	13.6
Grade 2	10	22.7
Grade 3	23	52.3
Not known	5	11.4
Site of metastases		
Lung	31	70.4
Liver	17	38.6
Soft tissue	17	38.6
Viscera	6	13.6
Bone	4	9.1
None	5	11.3
Number of metastatic sites		
0	5	11.3
1	16	26.4
2	13	29.5
3	8	18.2
4	2	4.5
Previous radiotherapy		
None	30	68.2
Pelvic	9	20.5
Other site	5	11.4
Previous surgery		
Yes	36	81.8
No	8	18.2

### Treatment and toxicity

The median number of cycles of study treatment per patient was 6 (range 2–8 cycles). Eleven patients (25.0%) received 2 cycles, 4 (9.1%) received 3 cycles, 4 (9.1%) received 4 cycles, 2 (4.5%) received 5 cycles, 13 (29.5%) received 6 cycles, 1 (2.3%) received 7 cycles, and 9 (20.5%) received 8 cycles. Nine patients required a dose reduction (25% in 8 patients, 50% in 1 patient). Of these, 8 patients started at 100% dose, and 1 patient at 75% dose due to previous pelvic radiotherapy.

Toxicities observed with the study treatment are summarised in Table 2. The commonest toxicities (any grade) were anaemia (95%), fatigue (93%), alopecia (88%), and thrombocytopenia (71%). The commonest grade 3 or 4 toxicities were fatigue (30%), anaemia (24%), dyspnoea (16%), neutropenia (12%), and infection (any, 12%). Eight patients (18%) stopped study treatment early specifically due to toxicity.

**Table 2:** Adverse events related to the study treatment

Adverse event	NCI common toxicity criteria grade					
	1	2	3	4	Any (%)	Total
Neutropenia	5	4	1	4	14 (30)	42
Thrombocytopenia	19	8	1	2	30 (71)	42
Anaemia	13	17	8	2	40 (95)	42
Infection	16	8	5	0	29 (67)	43
Vomiting	11	4	3	0	18 (43)	42
Diarrhoea	14	7	4	0	25 (58)	43
Dyspnoea	3	11	5	2	21 (49)	43
Fatigue	13	14	12	1	40 (93)	43
Neuropathy	19	2	0	0	21 (49)	43
Allergy	5	0	0	1	6 (14)	43
Alopecia	11	26	0	0	37 (88)	42
Joint pain	15	9	0	0	24 (56)	43

### Treatment response and survival

RECIST-measured objective PR were observed in 11 patients (25%), and 16 patients (36.4%) achieved confirmed SD. A further 7 patients (15.9%) had SD that was unconfirmed by RECIST, and 10 patients (22.7%) had disease progression. Comparing uterine versus non-uterine primary site, PR were seen in 8 (33%) and 3 (15%) patients, respectively, and confirmed SD in 7 patients (29.3%) and 8 (40%) patients, respectively.

**Figure 1:**
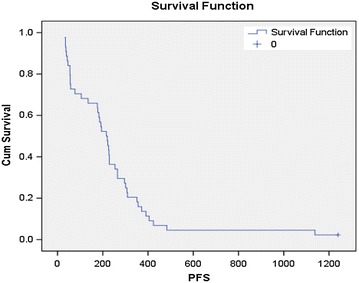
Kaplan–Meier curve of progression-free survival (days) for patients with advanced leiomyosarcoma (n = 44). Median progression free survival 7.1 months (95% CI 5.7–8.3 months).

At a median follow-up of 41 months, the median PFS was 7.1 months (95% confidence interval, CI, 5.7–8.3 months) (Figure 1), with progression-free rates at 3 and 6 months of 70.5% (95% CI 56.7–84.2%) and 59.1% (95% CI 44.3–73.9%), respectively. The median OS was 17.9 months (95% CI 10.6–25.2 months), with OS rates at 12 and 24 months of 65.5% (95% CI 51.1–79.9%) and 33.6% (95% CI 18.8–48.4%), respectively.

### Post-progression treatment

After treatment within the study, further treatment was as follows: 31 patients received second line systemic therapy (doxorubicin n = 31, ifosfamide n = 1, letrozole n = 1, pazopanib n = 1), 12 patients received third line systemic therapy (clinical trial n = 3, trabectedin n = 5, megesterol acetate n = 1, ifosfamide n = 2, gemcitabine and docetaxel n = 1), and 1 patient received fourth line systemic therapy (ifosfamide). Two patients underwent surgery (1 patient underwent palliative resection of residual pelvic disease, 1 patient underwent resection of a uterine mass that was deemed inoperable prior to chemotherapy), 2 patients received palliative radiotherapy alone, 6 patients had no further treatment, and information was missing for 2 patients.

## Discussion

Doxorubicin-based chemotherapy has remained the first line treatment for locally advanced/metastatic STS since its introduction nearly 40 years ago [3]. In that time no alternative regimen has been shown to be superior. However, there has been increasing recognition in recent years that it is no longer appropriate to consider all STSs as one homogeneous group, and that individual STS subtypes may respond differentially to different treatment regimens [14–16]. One of the earliest indications of using a specific treatment regimen in a STS subtype was the use of gemcitabine and docetaxel in leiomyosarcoma (Table 3). An initial study, predominantly in patients with uterine primary tumours, reported an ORR of 53% and median time to progression of 5.6 months [8]. The suggestion that there might be increased benefit for uterine leiomyosarcoma led to the investigation of the regimen in this patient group with phase II studies in the first [6] and second [5] line setting for advanced metastatic disease, with median PFS of 4.4 and 6.7 months, respectively. It has also been used in this patient cohort in the adjuvant setting in two phase II studies [17, 18], and has shown sufficient promise for it to now be studied in an international prospective randomised phase III study of adjuvant gemcitabine and docetaxel for 4 cycles followed by doxorubicin for 4 cycles, compared with observation for early stage resected uterine leiomyosarcoma (NCT01979393).

**Table 3:** Phase II studies of gemcitabine and docetaxel in locally advanced/metastatic leiomyosarcoma

Study	N^a^	uLMS^b^	LMS^c^	Line of treatment	Median PFS^d^ (months)	3 month PFR^e^ (%)	6 month PFR (%)
Hensley et al. [8]	34	✓	✓	1st and 2nd	5.6	–	47
Hensley et al. [6]	42	✓	–	1st	4.4	59.5	40.5
Hensley et al. [5]	48	✓	–	2nd	6.7+	73	52
Pautier et al. [7]	46	✓	✓	2nd	3.4–4.7^f^	53–71^f^	47–48^f^
This study	44	✓	✓	1st	7.1	70.5	59.1

^a^Number of subjects.

^b^Uterine leiomyosarcoma.

^c^Leiomyosarcoma.

^d^Progression-free survival.

^e^Progression-free rate.

^f^Results reported for uLMS and LMS separately.

Gemcitabine and docetaxel have been investigated in leiomyosarcoma as a specific group with the randomised phase II TAXOGEM study, which comparing with single agent gemcitabine for metastatic/relapsed disease in the second line setting [7]. It was stratified for uterine and non-uterine leiomyosarcoma, but was not able to demonstrate any difference between the two groups. Gemcitabine alone appeared to yield similar results to the combination but with less toxicity.

Our study was conceived following the publication of the first phase II study [8], with the aim of trying to replicate the early promising results, but in a cohort of leiomyosarcoma patients with locally advanced/metastatic disease in the first line setting. We have shown an ORR of 25%, with a 36.4% of patients achieving SD (with RECIST confirmation), giving a crude clinical benefit rate of 61.4%. A further 15.9% of patients had SD but without RECIST confirmation. The median PFS was 7.1 months, with a progression-free rate at 3 and 6 months of 70.5 and 59.1%, respectively. These results are consistent with the other studies summarised in Table 3. Patients went on to receive a range of treatments subsequent to trial participation, predominantly with further chemotherapy as would be expected in the context of metastatic disease.

Our patients received a median of 6 cycles of chemotherapy, and indeed only 20% completed the full 8 cycles. The commonest toxicities were fatigue and anaemia, experienced by >90% of patients. Dose reductions were required in 20% of patients, and treatment was stopped early in 18% specifically due to toxicity. Hence, the regimen as given in this study was relatively toxic, which needs to be borne in mind for chemotherapy being given in a non-curative, palliative setting.

The use of gemcitabine and docetaxel has been extended beyond leiomyosarcoma to STSs in general, and a randomised phase II study of 122 patients has compared the combination with gemcitabine alone [19], showing both regimens to be active, but with superior PFS for the combination (6.2 months) compared with gemcitabine alone (3.0 months). However, patients receiving the combination had a higher probability of stopping treatment early due to toxicity.

Despite the increasing evidence in the phase II setting to support the use of gemcitabine and docetaxel in STS, there has as yet been no phase III comparison with the current standard of care. We therefore decided to conduct a prospective randomised phase III study (GeDDiS) of gemcitabine and docetaxel compared with doxorubicin, in locally advanced/metastatic STS in the first line setting (CRUK/10/004). This study has been designed to compare efficacy and toxicity, and also includes health economic and quality of life data collection, in order to fully compare the two regimens. In view of the toxicity findings of our phase II study, we decided to use lower chemotherapy doses (75% of those used in the phase II study), and to limit treatment to 6 cycles. Recruitment has been conducted across the United Kingdom and Switzerland, and was completed with the accrual of 254 patients in January 2014. Initial results are anticipated in 2015.

## Conclusions

In conclusion, we have confirmed previous findings of the activity of gemcitabine and docetaxel in the first line setting in locally advanced/metastatic leiomyosarcoma, showing it to be a regimen worthy of further investigation. Our study has led to an international UK-based phase III study of gemcitabine and docetaxel compared with doxorubicin in locally advanced/metastatic STS, which has just completed recruitment. Results are awaited with interest to understand how this schedule fits into the current treatment algorithm for systemic treatment of STS.
